# The current evidence base for the feasibility of 48-hour continuous subcutaneous infusions (CSCIs): A systematically-structured review

**DOI:** 10.1371/journal.pone.0194236

**Published:** 2018-03-14

**Authors:** James Baker, Andrew Dickman, Stephen Mason, John Ellershaw

**Affiliations:** 1 Pharmacy Department, Royal Liverpool and Broadgreen University Hospitals NHS Trust, Liverpool, Merseyside, United Kingdom; 2 Palliative Care Institute Liverpool, University of Liverpool, Liverpool, Merseyside, United Kingdom; 3 Academic Palliative and End of Life Care Department, Royal Liverpool and Broadgreen University Hospitals NHS Trust, Liverpool, Merseyside, United Kingdom; Clinical Reader in Palliative Medicine and Medical Oncology, University of Nottingham, UNITED KINGDOM

## Abstract

**Background:**

A continuous subcutaneous infusion (CSCI) is an effective method of multiple drug administration commonly encountered in end of life care when the oral route is compromised. At present, current practice is to limit syringe driver infusion time to a maximum of 24 hours as dictated by available chemical stability data. However, the ability to deliver prescribed medication by a CSCI over 48 hours may have numerous benefits in both patient care and health service resource utilisation.

**Aim:**

To examine and present the current evidence base for the stability of 48-hour multiple-drug CSCIs in current clinical practice.

**Design:**

A systematically-structured review following PRISMA guidelines.

**Data sources:**

Three electronic databases and the grey literature were searched with no time limits. Empirical studies reporting data on the chemical stability of continuous subcutaneous infusions or solutions stored in polypropylene syringes were included.

**Results:**

Twenty-one empirical studies were included in this review reporting chemical compatibility and stability of 32 discrete combinations of twenty-four drugs tested at a variety of different drug concentrations. The majority of combinations reported were assessed as being chemically compatible. The greatest risk of clinically significant chemical degradation was observed with midazolam. Only one study reported the microbiological stability of the solution examined.

**Conclusions:**

There is currently limited evidence for the physical, chemical and microbiological stability of solutions for continuous subcutaneous infusion over a period of 48 hours. More stability data is required before the use of 48-hour CSCIs can be evaluated for use within clinical practice.

## Introduction

With one third of all patients in UK District General Hospitals expected to be in last year of life, end-of-life care is considered one of the key domains of care[1]. The challenge in providing appropriate end-of-life care is daunting; in 2014, 468,875 deaths were recorded in the UK[2] and this is projected to rise approximately 20% to 561,000 deaths per year by 2035/36[3]. The Department of Health has committed to developing personalised care for people approaching the end of life and to improve care quality across all healthcare settings[4].

A continuous subcutaneous infusion (CSCI) is an effective method of multiple drug administration commonly encountered in end of life care when the oral route is compromised[5, 6]. In the United Kingdom, current practice for the administration of drugs by CSCI is to limit infusion time to a maximum of 24-hours, as a result of the limited availability of chemical and microbial safety data. This has been acknowledged by the Commission on Human Medicines (CHM) who have proposed that research to develop authoritative national guidance be commissioned, and the National Patient Safety Agency’s recommendation that ward-prepared infusions be limited to 24-hour infusion duration[7].

In contrast to UK clinical practice, Spanish Palliative Care centres regularly infuse multiple-drug containing CSCIs to patients over a 5- to 7-day period via either a syringe pump or PVC cassette[8]. This, in addition to the results of a recent evaluation of CSCI prescribing across seven NHS acute hospitals in the North of England which found that the median frequency at which medication changes (dosage or combination) were made to a CSCI prescription was 2 days, suggests that there is potential to change UK clinical practice from 24 to 48-hourly infusions[9]. The increase in infusion duration may allow clinical staff more time to focus on compassionate care and additionally benefit health service resource utilisation.

Before a clinical trial can be performed to assess the acceptability, efficacy and impact of 48-hour CSCIs in the United Kingdom, robust chemical and microbiological stability data of 48-hour CSCI drug combinations are required to ensure patient safety. This systematically-structured review aims to identify and review the current evidence base for the chemical compatibility and stability of multiple-drug solutions used for symptom relief either undergoing infusion or storage in polypropylene syringes.

## Methods

### Procedure

The search strategy was structured in concordance with PRISMA guidelines (http://www.prisma-statement.org) and a review protocol developed and internally peer reviewed. Three electronic databases (CINAHL, MEDLINE and EMBASE) were searched for articles published with no time limits using the search strategy described in Table 1. Search terms were developed in relation to the aims, and MeSH headings were used where available. Inclusion criteria comprised of: peer-reviewed articles examining the physical and chemical compatibility of multi-drug 48-hour syringe drivers (Table 2); stability and compatibility reported using a validated analytical technique, such as high-performance liquid chromatography (HPLC)[10]. For this review, the term “Drugs used in CSCI/syringe indicated for use in palliative care CSCIs” was defined as drugs with established use in UK palliative care practice for the relief of commonly-encountered symptoms (e.g. nausea, pain, shortness of breath etc). The most recent survey of UK CSCI prescribing practice[11] was referred to for a list of commonly prescribed drugs.

**Table 1:** Search strategy and results of each search (Capital letters = term from database thesaurus i.e. MeSH).

Search Terms	CINAHL	MEDLINE	EMBASE
**1. ("Continuous Subcutaneous Infusion" OR “Syringe driver” OR “Syringe Pump” OR “CSCI” or “Syringe”).af;**	12,500	16,917	23,812
**2. (“Drug stability” OR “Chemical Stability”).af;**	1,006	44,336	65,564
**3. exp “CHROMATOGRAPHY, HIGH PRESSURE LIQUID”/**	3,507	166,606	276,215
**4. (“HPLC” OR “High Pressure Liquid Chromatography”).af**	5,131	158,424	307,244
**5. 3 OR 4;**	7,181	236,424	317,363
**6. (“Palliative Care” OR “End of Life Care”).af**	61,004	61,014	49,362
**7. (1 AND 2 AND 5 AND 6)**	7	8	16

**Table 2:** Inclusion and exclusion criteria for systematic search.

Inclusion	Exclusion
Experimental study design—stability/compatibility confirmed using validated analytical techniques	Observational study design—physical stability only confirmed by observing for precipitation
CSCI/Syringe investigated for 48-hour duration or longer	CSCI/Syringe investigated for less than 48 hours
CSCI/Syringe investigated contained at least two drugs	CSCI/Syringe investigated contained only one drug
Drugs in CSCI/Syringe indicated for use in palliative care CSCIs	Drugs in CSCI/Syringe not indicated for use in palliative care CSCIs

As additional steps to ensure maximal data capture, the bibliographies of articles selected for full-text review were examined with the aim of identifying additional articles. A “grey literature” search was performed using Google and Google Scholar in an attempt to discover as many published and unpublished studies as possible for inclusion in this review.

**Fig 1:**
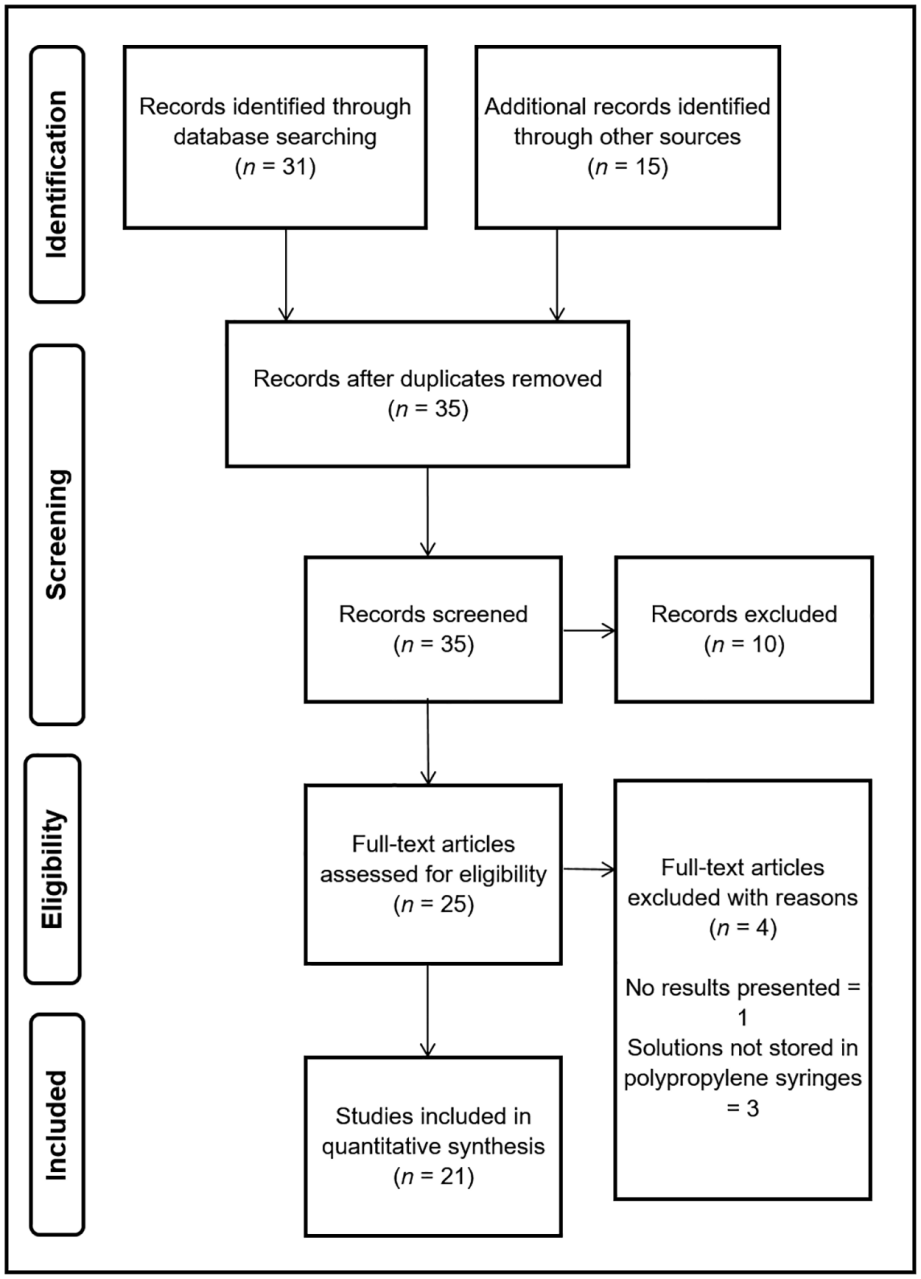
PRISMA flowchart.

Titles and abstracts of all articles across the steps outlined in the PRISMA process (Fig 1) were screened by two reviewers (JB+AD) to identify papers that met the study’s inclusion criteria. The articles included from the abstract stage were read in full and their reference lists searched by JB and AD with any queries regarding a papers eligibility were resolved following a discussion between JB, AD and SM. Articles from this stage that met the study’s criteria proceeded to full-text assessment. Full-text assessment was performed using a data extraction tool adapted from Hawker et al[12].

## Results

The initial search of databases yielded 31 results: EMBASE (*n* = 16), CINAHL (*n* = 7), MEDLINE (*n* = 8), this reduced to 20 articles following removal of duplicates. On the basis of title or abstract, 7 articles were excluded leaving 13 articles for full-text assessment. 4 articles meeting the inclusion criteria were identified from the grey literature. 11 additional articles were further identified following a hand-search of the reference list of eligible papers. Of the 15 articles identified from the grey literature and hand-search of reference lists, 3 articles were excluded on basis of abstract. This left 25 articles in total for full-text assessment, which resulted in a further 4 articles being excluded for not meeting eligibility criteria. This left 21 papers which reported chemical stability and compatibility data for multiple-drug containing syringes over a 48-hour or greater time period (Fig 1).

### Study characteristics, design and quality

Twenty-one empirical studies were included in this review. As methodology varied greatly across the included studies, as did the drug combinations investigated, a meta-analysis could not be performed (S1 Table).

The majority (*n* = 12) of the articles were published from 2000–2010, with the remainder being published either before 2000 (*n* = 8) or after 2010 (*n* = 1). Five of the studies included were performed in Spain and the United Kingdom, four undertaken in Australia and Canada, with the remaining three undertaken in Finland, Singapore and Italy.

All studies recorded both qualitative analysis of physical stability/compatibility (visual observation for precipitation/degradation) and quantitative analysis of chemical stability (concentration determination through High Performance Liquid Chromatography (HPLC)). Quantitative data was analysed in a variety of ways across the 21 studies, with 3 employing two-way analysis of variance with replicates[13–15], 2 employing regression analysis[16, 17], 15 employing the mean of multiple replicates with standard deviations[18–32]. One study employed no form of statistical analysis[33].

Eighteen of the included studies investigated compatibility at ambient temperature (18–25°C)[13–21, 25–29, 31–34], eleven also investigated compatibility at refrigerated temperature (4–8°C)[15, 18–21, 25–28, 31, 32], five investigated at body temperature (36–38°C)[13, 15, 25, 32, 35] and two studies investigated compatibility at 32°C, simulating a syringe held in a sling close to the patients’ body[24, 30].

### Drugs and doses investigated

A total of 24 drugs were included in the 21 studies, involving 32 different combinations. The most drugs included in a single combination were six[26] and the least included in a single combination were two[13, 15–21, 23, 24, 28, 29, 32–34]. The drugs included most frequently in syringe driver combinations reported were haloperidol (*n =* 13), morphine sulphate, midazolam and dexamethasone (*n* = 7 each). The most commonly included opioid was morphine, with 7 combinations including the sulphate salt, 3 including the hydrochloride salt and 1 including morphine tartrate. Fifteen studies reported using sodium chloride 0.9% as a diluent, two studies reported using Water for Injections and five studies report using no diluent. Table 3 outlines the drug combinations reported and the dose ranges used.

**Table 3:** Summary of drug dosage and combinations reported.

Study, Year and Country	Drug combination and concentration range	Dose range (mg)	Final Volume (mL)	Drug Concentration range (mg/mL)	Diluent	Incompatibility observed by 48 hours?
**Good et al; 2004; Australia**[13]	Midazolam hydrochloride	2.5–7.5	8	0.3–0.9	NS	Yes, @37°C
	Dexamethasone sodium phosphate	2–4		0.25–0.5		
**Wilson et al; 1998;Australia**[15]	Fentanyl citrate	0.1–0.6	8 or 18	0.0125–0.033	NS	Yes @ 37°C
	Midazolam hydrochloride	5–15		0.28–0.93		
**Negro et al;2006; Spain**[14]	Morphine hydrochloride	100–600	60	1.67–10	NS	No
	Haloperidol lactate	25–37.5		0.417–0.625		
	Hyoscine-N-butylbromide	300–400		5–6.67		
**Peterson et al; 1998; Australia**[30]	Fentanyl citrate	1	24.5	0.04	Nil	No
	Hyoscine-N-butylbromide	30		1.22		
	Midazolam hydrochloride	15		0.61		
	Fentanyl citrate	1	27	0.037	Nil	No
	Metoclopramide hydrochloride	20		0.74		
	Midazolam hydrochloride	15		0.56		
**Barcia et al; 2003; Spain**[19]	Haloperidol lactate	18.75–75	60	0.31–1.25	NS	Yes, @ 4°C and 25°C
	Hyoscine-N-butylbromide	150–600		2.5–10		
**Targett et al; 1997; Australia**[31]	Morphine tartrate	40–400	10	4–40	NS	Yes @ 22°C
	Dexamethasone sodium phosphate	8		0.8		
	Droperidol	2		0.2		
	Hyoscine-N-butylbromide	20		2		
	Midazolam hydrochloride	5–8		0.5–0.8		
**Fielding et al; 2000; UK**[35]	Diamorphine hydrochloride	50–200	8	12.5–25	WFI	No
	Octreotide acetate	0.3–0.9		0.375–1.5		
**Negro et al; 2007; Spain**[29]	Tramadol hydrochloride	500–2000	60	8.33–33.33	NS	No
	Dexamethasone sodium phosphate	20–200		0.33–3.33		
**Destro et al; 2012; Italy**[33]	Morphine hydrochloride	20–100	17	1.18–5.88	NS	Yes @ 25°C
	Ketorolac tromethamine	30–90		1.76–5.29		
**Grassby et al; 1997; UK**[17]	Diamorphine hydrochloride	6–99	1	6–99	Nil	Yes @ 22°C
	Cyclizine lactate	4–52		4–52		
	Diamorphine hydrochloride	20–100	1	20–100	Nil	No
	Haloperidol lactate	2–4		2–4		
	Diamorphine hydrochloride	11–56	1	11–56	Nil	No
	Cyclizine lactate	9–27		9–27		
	Haloperidol lactate	2.1–2.4		2.1–2.4		
**Negro et al; 2006; Spain**[28]	Furosemide sodium	200–600	60	3.33–10	NS	No
	Dexamethasone sodium phosphate	20–200		0.33–3.33		
**Nassr et al; 2003; Canada**[27]	Hydromorphone hydrochloride	50	5	10	NS	No
	Midazolam hydrochloride	5		1		
	Famotidine	2		0.4		
	Hydromorphone hydrochloride	50	5	10	NS	Yes @ 4°C
	Metoclopramide hydrochloride	2.5		0.5		
	Haloperidol lactate	2.5		0.5		
	Hydromorphone hydrochloride	50	5	10	NS	No
	Ketorolac tromethamine	7.5		1.5		
	Metoclopramide hydrochloride	2.5		0.5		
	Famotidine	2		0.4		
	Hydromorphone hydrochloride	50	5	10	NS	Yes @ 4°C
	Dimenhydrinate	25		5		
	Haloperidol lactate	2.2		0.5		
	Famotidine	2		0.4		
	Hyoscine hydrobromide	0.2		0.04		
**Nassr et al; 2001; Canada**[26]	Morphine sulphate	50	5	10	NS	No
	Dexamethasone sodium phosphate	2		0.4		
	Octreotide acetate	0.05		0.01		
	Morphine sulphate	50	5	10	NS	Yes @ 4°C and 25°C
	Dexamethasone sodium phosphate	2		0.4		
	Haloperidol lactate	2.5		0.5		
	Morphine sulphate	50	5	10	NS	Yes @ 25°C
	Octreotide acetate	0.05		0.01		
	Haloperidol lactate	2.5		0.5		
	Midazolam hydrochloride	5		1		
	Famotidine	2		0.4		
	Morphine sulphate	50	5	10	NS	No
	Haloperidol lactate	2.5		0.5		
	Famotidine	2		0.4		
	Metoclopramide hydrochloride	2.5		0.5		
	Morphine sulphate	50	5	10	NS	No
	Octreotide acetate	0.05		0.01		
	Haloperidol lactate	2.5		0.5		
	Famotidine	2		0.4		
	Metoclopramide hydrochloride	2.5		0.5		
	Dimenhydrinate	25		5		
**Donnelly; 2009; Canada**[21]	Morphine sulphate	60–300	30	2–10	NS	No
	Ketamine hydrochloride	60		2		
**Ensom et al; 2009; Canada**[34]	Hydromorphone hydrochloride	10	50	0.2	NS	No
	Ketamine hydrochloride	10–50		0.2–1		
**Watson et al; 2005; UK**[32]	Dexamethasone sodium phosphate	1	14	0.07	NS	No
	Ketamine hydrochloride	50–600		3.57–12		
**Hor et al; 1997; Singapore**[24]	Pethidine hydrochloride	50	3	16.7	Nil	No
	Metoclopramide hydrochloride	10		3.33		
**Jäppinen et al; 1999; Finland**[25]	Buprenorphine hydrochloride	4	48	0.084	NS	No
	Haloperidol lactate	5		0.104		
	Glycopyrronium bromide	1.2		0.025		
**Barcia et al; 2005; Spain**[18]	Morphine hydrochloride	100–600	60	1.67–10	NS	No
	Hyoscine-*N*-butylbromide	200–400		3.33–6.67		
**Allwood; 1991; UK**[16]	Diamorphine hydrochloride	20–200	10	2–20	WFI	No
	Haloperidol lactate	7.5		0.75		
	Diamorphine hydrochloride	20–200	10	2–20	WFI	No
	Cyclizine lactate	67		0.67		
**Collins et al; 1990; UK**[20]	Diamorphine hydrochloride	50–100	8	6.25–13	WFI	No
	Haloperidol lactate	2.5		0.31		

Key: NS—Sodium chloride 0.9%; WFI—Water for Injections; Nil—No diluent used.

### Methods used to investigate stability and compatibility

All the included studies used HPLC to confirm the concentration of the drugs in the syringe driver at each time point of assessment according to each study’s methodology. All studies reported using analytical grade compounds in the generation calibration curves and were linear with coefficients of determination (r^2^) greater than 0.999 for the concentration of solutions tested.

All studies also reported visually examining the samples at each time point of analysis, as determined by the design of each included article, for signs of physical instability such as crystallisation, precipitation or evaporation. All studies also tested the pH of samples both pre- and post- the analytical period to observe for any variation in addition to the HPLC testing. All studies, bar one (Destro et al [33]), as a minimum used a mean of replicate results and standard deviation or better for analysing the chemical compatibility and stability of the drug combination investigated by HPLC. One study[25] investigated the microbiological stability of the solutions tested over a 30-day period using the technique of membrane filtration to European Pharmacopoeia standards.

### Compatibility of drug combinations reported

#### Dexamethasone and midazolam

Good et al[13] investigated the compatibility and stability of midazolam 2.5mg, 5mg or 7.5mg and dexamethasone 2mg or 4mg made up to a final volume of 8mL using sodium chloride 0.9% and stored in 10mL polypropylene syringes at 22–26°C or 35–39°C for 48 hours. Syringes containing midazolam 5mg or 7.5mg and dexamethasone 4mg were not analysed due to the solutions becoming cloudy immediately upon preparation, suggesting chemical incompatibility. It is presumed that the incompatibility between midazolam and dexamethasone is due to the alkaline nature of dexamethasone causing an increase in solution pH. This pushes the pH dependent equilibrium of midazolam towards the closed-ring, more lipophilic structure, resulting in reduced solubility[36]. Syringes containing midazolam 7.5mg and dexamethasone 2mg stored at 37°C showed signs of crystallisation; HPLC analysis indicated a clinically significant decrease (defined by Trissel[37] as ±10% of drug) in midazolam concentration. Replicate syringes subjected to identical storage and temperature procedures produced no visible crystallisation, but HPLC analysis again indicated a clinically significant decrease in midazolam concentration.

Of the remaining mixtures, no signs of physical incompatibility were observed, however, only the combinations of midazolam 5mg with dexamethasone 2mg, and midazolam 7.5mg with dexamethasone 2mg retained a clinically significant midazolam concentration after 48 hours at room temperature (22–26°C). No mixtures investigated retained a clinically significant midazolam concentration when stored at 37°C.

#### Fentanyl and midazolam

Wilson et al[15] investigated the compatibility and stability of fentanyl 100 micrograms, 300 micrograms or 600 micrograms in combination with midazolam 5mg, 7.5mg or 15mg, diluted to either 8 or 18ml with sodium chloride 0.9% and stored in 10 or 20ml polypropylene syringes at 21–23°C, 3–7°C and 37–39°C for seven days. No physical incompatibility was noted for any admixture throughout the investigation. Neither time nor temperature affected the stability of fentanyl at any concentration, but midazolam appeared to be subject to time-dependent degradation, especially at higher temperatures. However, clinically significant degradation (12.1% in the mixture containing fentanyl 300 micrograms/midazolam 7.5mg and 11.7% in the mixture containing fentanyl 600 micrograms/midazolam 5mg) was only reported after 7 days in two of the six mixtures stored at 37–39°C.

#### Hyoscine-N-butylbromide and haloperidol

Barcia et al[19] investigated the chemical stability and physical compatibility of haloperidol 18.75mg, 37.5mg or 75mg in combination with hyoscine 150mg, 300mg or 600mg, diluted to 60mL with sodium chloride 0.9% and stored in polypropylene syringes at 4°C or 25°C over a period of 15 days. Precipitation was reported in syringes containing haloperidol 75mg and hyoscine-*N-*butylbromide 150mg, 300mg or 600mg stored at 4°C after 7 days and slight precipitation at 25°C after 15 days. Analysis of precipitates found that they contained haloperidol. HPLC analysis of all solutions showed no clinically significant loss of hyoscine-*N*-butylbromide or haloperidol throughout the duration of investigation.

#### Morphine tartrate, dexamethasone, droperidol, hyoscine-N-butylbromide and midazolam

Targett et al [22] investigated the chemical stability and physical compatibility of two different formulations of morphine tartrate in combination with dexamethasone, droperidol, hyoscine-*N*-butylbromide and midazolam, diluted to 10ml with sodium chloride 0.9% in 10ml polypropylene syringes, stored at 4–8°C and 21–23°C. The first formulation was comprised of morphine tartrate 400mg, dexamethasone 8mg, droperidol 2mg, hyoscine-*N*-butylbromide 20mg and midazolam 8mg. The second formulation was comprised of morphine tartrate 40mg, dexamethasone 8mg, droperidol 2mg, hyoscine-*N*-butylbromide 20mg and midazolam 5mg. Physical compatibility was observed with the solution containing the lower concentration or morphine tartrate (4mg/mL) at either temperature, but the development of a slight yellow colour in formulations containing the higher concentration of morphine tartrate (40mg/mL) at ambient (21–23°C) temperature was reported; this was thought to be due to the polymerisation of morphine degradation product[38]. The concentration of midazolam was observed to have decreased to a clinically significant proportion after 12 days in syringes containing 40mg/mL morphine tartrate stored at 21–23°C, and after 5 days in syringes containing 4mg/mL morphine tartrate stored at 21–23°C. No other clinically significant degradation of drugs was observed at either temperature throughout the period of investigation.

#### Morphine hydrochloride and ketorolac

Destro et al[33] investigated the physical and chemical compatibility of morphine hydrochloride and ketorolac tromethamine at a range of concentrations over a 48-hour time period. Morphine was found to be physically and chemically incompatible with both ketorolac 1.76mg/mL at morphine concentrations above 5.88mg/mL and ketorolac concentration 3.53mg/mL at morphine concentrations above 4.7mg/mL. Morphine hydrochloride was also found to be physically incompatible with ketorolac concentrations above 5.29mg/mL. On analysis using HPLC techniques, degradation of both morphine hydrochloride and ketorolac tromethamine was observed.

Destro et al also investigated physical compatibility between binary mixtures of morphine hydrochloride with diclofenac sodium, methadone hydrochloride with ketoralac tromethamine, and methadone hydrochloride with diclofenac sodium. All mixtures containing varying concentrations of these drugs were found to develop a precipitate upon mixing and were not analysed by HPLC. However, precipitate formed upon the mixing of ketorolac and methadone dissolved upon being diluted to volume with sodium chloride 0.9%.

#### Diamorphine hydrochloride and haloperidol

Allwood[16] investigated the compatibility and stability of diamorphine hydrochloride in combination with haloperidol over a period of 45 days. Diamorphine was found to be compatible with haloperidol 0.75mg/mL between concentration ranges of 20mg/mL to 100mg/mL at ambient temperatures following both visual inspection and HPLC analysis. The combination of diamorphine hydrochloride 20mg and haloperidol 7.5mg, diluted to 10ml with water for injections BP and stored in a 10mL syringe, was stable for at least 14 days; and the combination of diamorphine hydrochloride 200mg with haloperidol 7.5mg remained stable for at least 20 days when stored at ambient temperature. Grassby & Hutchens[17] identified no instability when binary combinations of diamorphine and haloperidol, at concentrations between 20-100mg/mL and 2-4mg/mL respectively, were stored at room temperature for seven days.

#### Diamorphine hydrochloride and cyclizine

Allwood[16] also investigated the compatibility and stability of diamorphine hydrochloride in combination with haloperidol over a period of 45 days. Diamorphine was found to be incompatible with cyclizine 10mg/ml at concentrations above 25mg/mL when stored at ambient temperatures; it was however determined that cyclizine 6.7mg/mL was compatible with diamorphine hydrochloride at all concentrations up to 100mg/mL. The combination of diamorphine hydrochloride 20mg and cyclizine 67mg, diluted to 10mL with water for injections BP and stored in a 10ml syringe, was stable for at least 13 days; and the combination of diamorphine hydrochloride 200mg with cyclizine 67mg remained stable for at least 9 days when stored at ambient temperature. Grassby & Hutchens[17] reported that the incompatibility observed when mixing diamorphine and cyclizine is due to the formation of cyclizine hydrochloride crystals. The formation of these crystals is dependent on the relative and total concentration of cyclizine lactate, diamorphine hydrochloride and other chloride ions in the mixture.

#### Hydromorphone, metoclopramide and haloperidol

Nassr et al[27] investigated the compatibility and stability of a tertiary combination of hydromorphone 10mg/mL, metoclopramide 0.5mg/mL and haloperidol 0.5mg/mL, stored for four days at temperatures of 4 °C and 22 °C. No issues were observed with the syringes stored at 22 °C, but formation of precipitate on the surface of polypropylene syringes stored at 4 °C were observed after 12 hours. The precipitate was identified as being haloperidol through matching both the retention time and UV spectra of haloperidol and the precipitate when analysed using HPLC.

#### Hydromorphone, dimenhydrinate, haloperidol, famotidine and hyoscine hydrobromide

Nassr et al[27] investigated the compatibility and stability of a quintenary combination of hydromorphone 10mg/mL, dimenhydrinate 5mg/mL, haloperidol 0.5mg/mL, famotidine 0.4mg/mL and hyoscine hydrobromide 0.04mg/mL, stored for four days at temperatures of 4 °C and 22 °C. Physical incompatibility was observed with syringes stored at both temperatures, where a white precipitate was observed to form after 12 hours on the wall of the propylene syringe. Through HPLC analysis, the precipitate was identified as dimenhydrinate.

#### Morphine sulphate, dexamethasone and haloperidol

Nassr et al[26] attempted to investigate the compatibility and stability of a tertiary combination of morphine sulphate 10mg/mL, dexamethasone 0.4mg/mL and haloperidol 0.5mg/mL. However, it was noted that upon mixing of haloperidol and dexamethasone a white, fluffy precipitate was formed regardless of the order the drugs were mixed in.

#### Morphine sulphate, octreotide, haloperidol, midazolam and famotidine

Nassr et al[26] investigated the compatibility and stability of a quintenary combination of morphine sulphate 10mg/mL, octreotide 0.01mg/mL, haloperidol 0.5mg/mL, midazolam 1mg/mL and famotidine 0.4mg/mL. Physical incompatibility was observed after 24 hours in the syringes stored at 4 °C where a precipitate was noted to form on the surface of the polypropylene syringe. The precipitate was observed to be of crystalline and transparent in nature, and was identified as being haloperidol following HPLC analysis.

## Discussion

The systematically structured review has identified that there is currently limited 48-hour chemical compatibility and stability data published for the most common CSCI combinations used in the UK, as determined by the most recent analysis of national CSCI prescribing practice[11]. All the included articles investigated the compatibility and physicochemical stability of pre-mixed multi-drug polypropylene syringes stored at a range of different temperatures over extended time periods, with no evidence of their use or impact in current clinical practice.

### Microbiological and chemical compatibility

Midazolam is the benzodiazepine of choice for use in the CSCIs of patients suffering from terminal agitation, and this is reflected in a national survey identifying midazolam as the most-frequently prescribed drug in a CSCI[11]. All studies included that used midazolam reported potential stability issues over extended time periods, with stability decreasing with increasing pH due to the pH-dependent equilibrium of midazolam’s ring structure. The reduction in midazolam concentration at 48 hours, for the majority of midazolam containing CSCI combinations stored at room temperature included in this review, was not clinically significant. However four of the ten concentrations tested by Good et al showed a clinically significant reduction in midazolam concentration at 48 hours when stored at body temperature (35–39°C)[13]. All combinations affected were binary admixtures of midazolam hydrochloride (ampoule pH 3.5) and dexamethasone sodium phosphate (ampoule pH 7), with the reduced stability due to the alkaline nature of the dexamethasone component causing an increase in the presence of the more lipophilic (and therefore less water soluble) closed-ring structure midazolam molecule[36].

Hyoscine-*N*-butylbromide, haloperidol, diamorphine and fentanyl appear to be physicochemically stable at concentrations investigated in the articles included in this review and are broadly comparable to those encountered in 24-hour CSCIs in the UK[11]. However, for a 48-hour CSCI the concentrations may require to be doubled. Barcia et al[19] investigated haloperidol 1.25mg/mL with hyoscine-*N*-butylbromide 10mg/mL, concentrations equivalent to 15mg/day haloperidol and hyoscine-*N*-butylbromide 120mg/day (double the usual clinical dose) providing some evidence for the stability of these two drugs combined in a 48 hours CSCI.

### Relevance of data for clinical practice in the UK

Of the twenty-four drugs used by the studies included in the review, twelve are regularly utilised in the prescribing of CSCIs in the United Kingdom (cyclizine, dexamethasone, diamorphine, fentanyl, glycopyrrolate, haloperidol, hyoscine hydrobromide, hyoscine-*N-*butylbromide, metoclopramide, midazolam, morphine sulphate and octreotide) [37]. Furosemide, hydromorphone ketamine, ketorolac and tramadol, are occasionally, but uncommonly used in UK clinical practice. Morphine hydrochloride and morphine tartrate are not licensed for use in the UK, however morphine as the sulphate salt is widely used. Buprenorphine and droperidol are not frequently used in the UK even though licensed preparations are available.

In the United Kingdom, the National Institute for Health and Clinical Excellence recommends that the parenteral opioid with the lowest acquisition cost should be used first-line[39]; for the UK this is currently morphine sulphate. Seven of the included studies investigated the stability and compatibility of this morphine salt with a further four investigating alternative salts, such as morphine tartrate or morphine hydrochloride. However, extrapolation of the data for these salts would not be suitable.

In the United Kingdom, CSCIs are infused using the CME Medical T34 syringe pump, which is typically used in combination with either a 20mL, or 30mL plastic syringe, limiting the maximum infusion volume available to 30mL. This is usually a sufficient volume for a 24-hour CSCI containing most multiple drug combinations. However, for a 48-hour CSCI infusion, volume could be an issue with some medications, such as fentanyl. Licenced preparations of fentanyl citrate for injection in the UK are 50 micrograms/mL. With the typical 24-hour dose being 500 micrograms/day, the volume required for sufficient fentanyl to be administered a 48-hour CSCI (20mL) would near the maximum volume able to be held by the largest available syringe before any other medications are added. The manufacturer of the T34 syringe pump has recently developed a new, larger, lockbox which will enable syringes of up to 50mL volume to be used with the T34, providing greater options for increased duration infusions of combinations found to be chemically compatible and stable. However, this treatment option may be unacceptable to some patients due to the increased bulk of the infusion device as a result of the larger syringe.

Taking the above into account, of the 32 combinations investigated, 20 included drugs at concentrations that are relevant to current UK practice. Current practice in Spain is the infusion of a 60mL syringe or medication cassette over a period of five days and is reflected in the experimental design of the Spanish studies included in this review[14, 18, 19, 28, 29]. However, the final concentrations of drugs in the admixtures were equivalent to that expected in most CSCI combinations in the UK.

The ability to administer CSCIs to patients over extended time periods may benefit patients through providing greater independence, autonomy and improved quality of life as a result of receiving a reduced number of interventions (such as syringe changes). However, the administration device and syringe in situ may still need to be checked (e.g. running to time/battery level/no signs of crystallization, etc.) in addition to the need to clinically assess patient and their carers. As a result of these unknown factors, any future investigation into the clinical utility of extended-duration continuous subcutaneous infusions should aim to identify and develop a model of care which mitigates the concerns of all stakeholders in such an intervention.

### Strength and limitations of the review

The search strategy employed in this review utilised the key-word functionality of the electronic databases searched (for example MeSH terms were identified and utilised in the searching of the MEDLINE database). This meant that the searches performed should have been broad enough to identify much of papers available; further to this the bibliographies of included articles were reviewed to identify any articles missed during the search of the electronic databases.

A limitation of this review is that additional drug combinations may have been tested but not published in peer-reviewed journals because of unexpected or negative results. Publication bias such as this may have hindered the literature search performed from identifying all available data.

## Conclusion

Given the current pressures on healthcare resources in the United Kingdom and further afield, innovation in how current clinical services are delivered is essential. This review aimed to evaluate the current evidence base to support increasing CSCI infusion times above the current maximum of 24 hours. However, as described above, there is currently insufficient evidence for the physical, chemical and microbiological stability of solutions for continuous subcutaneous infusion over a period of 48 hours. Using data from the most-recent analysis of CSCI prescribing in the United Kingdom[11], which has identified the most commonly prescribed drug/dose combinations, investigators can extrapolate the typical drug concentration and volume required for 48-hourly infusions for combinations which have no stability data identified in this systematically-structured review.

This systematic search of three electronic databases yielded no investigations examining the chemical stability of mixtures whilst undergoing simulated infusion—this technique would closely mimic clinical practice and not only produce results from admixture compatibility whilst in polypropylene syringes, but also during administration via polyvinylchloride giving sets.

Therefore, there is an opportunity and scope for the generation of 48-hour (or greater) stability data for the most frequently used CSCI combinations to allow investigations into the clinical utility of increased-duration CSCIs by clinicians globally.

A final theme identified by this review is that the range of temperatures at which stability is tested is variable, due to the range of environments in which patient’s store the medical device during infusion. This highlights the need for consensus on how stability/compatibility testing should be structured.

## Supporting information

S1 Table Summary of included articles. Summary table or articles included in this review.
(PDF)

S1 File Full text assessment form. Full text assessment form used, adapted from Hawker et al, 2002.
(PDF)

S2 File Reference 9 –Poster presented at APM Supportive and Palliative Care Conference. Poster presented by author at the APM Supportive and Palliative Care Conference 2017 and referenced in this review.
(PDF)

S3 File PRISMA Checklist. PRISMA Checklist for manuscript.
(PDF)

## References

[1] Clark D, Armstrong M, Allan A, Graham F, Carnon A, Isles C. Imminence of death among hospital inpatients: Prevalent cohort study. Palliative Medicine. 2014 10.1177/0269216314526443 24637342 PMC4845030

[2] Office for National Statistics. Deaths Registered by Area of Usual Residence, UK, 20142016 15/07/2016. https://www.ons.gov.uk/peoplepopulationandcommunity/birthsdeathsandmarriages/deaths/datasets/deathsregisteredbyareaofusualresidenceenglandandwales.

[3] Office for National Statistics. 2014-based National Population Projection: Principal Projection—England Summary.2015 15/07/2016. www.ons.gov.uk/peoplepopulationandcommunity/populationandmigration/populationprojections/datasets/tablea14principalprojectionenglandsummary.

[4] NHS Finance and Operations. Our Commitment to you for end of life care: The Government Response to the Review of Choice in End of Life Care. In: Department Of Health, editor. London: DOH; 2016.

[5] Ellershaw J, Ward C. Care of the dying patient: the last hours or days of life. BMJ. 2003;326(7379):30–4. PMC1124925 12511460

[6] Masman AD, van Dijk M, Tibboel D, Baar FP, Mathot RA. Medication use during end-of-life care in a palliative care centre. Int J Clin Pharm. 2015;37(5):767–75. 10.1007/s11096-015-0094-3 . 25854310 PMC4594093

[7] National Patient Safety Agency (NPSA). Promoting safer use of injectable medicines 2007 [cited 2016 14/09/2016]. http://www.nrls.npsa.nhs.uk/resources/?entryid45=59812

[8] Negro S, Azuara M, Sánchez Y, Reyes R, Barcia E. Physical compatibility and in vivo evaluation of drug mixtures for subcutaneous infusion to cancer patients in palliative care. Supportive Care in Cancer. 2002;10(1):65–70. 10.1007/s005200100303 11777190

[9] Baker J, Dickman A, Mason S, Jackson R, Bickerstaff M, Skipper P, et al. The frequency at which doses and drugs administered by continuous subcutaneous infusion are changed: a service evaluation of clinical practice in 7 Acute NHS Hospitals in Northern England. Poster presented at: The APM Supportive and Palliative Care Conference; 30–31 March 2017; Belfast30-31 March 2017.

[10] Blessy M, Patel RD, Prajapati PN, Agrawal YK. Development of forced degradation and stability indicating studies of drugs—A review. Journal of Pharmaceutical Analysis. 2014;4(3):159–65. 10.1016/j.jpha.2013.09.003. 29403878 PMC5761119

[11] Dickman A, Bickerstaff M, Jackson R, Schneider J, Mason S, Ellershaw J. Identification of drug combinations administered by continuous subcutaneous infusion that require analysis for compatibility and stability. BMC Palliative Care. 2017;16(1):22 10.1186/s12904-017-0195-y 28335763 PMC5364569

[12] Hawker S, Payne S, Kerr C, Hardey M, Powell J. Appraising the evidence: reviewing disparate data systematically. Qual Health Res. 2002;12 10.1177/1049732302238251 12448672

[13] Good PD, Schneider JJ, Ravenscroft PJ. The compatibility and stability of midazolam and dexamethasone in infusion solutions. Journal of Pain and Symptom Management. 2004;27(5):471–5. 10.1016/j.jpainsymman.2004.02.002. 15120775

[14] Negro S, Reyes R, Azuara ML, Sánchez Y, Barcia E. Morphine, haloperidol and hyoscine N-butyl bromide combined in s.c. infusion solutions: Compatibility and stability: Evaluation in terminal oncology patients. International Journal of Pharmaceutics. 2006;307(2):278–84. 10.1016/j.ijpharm.2005.10.017. 16297583

[15] Wilson KM, Schneider JJ, Ravenscroft PJ. Stability of Midazolam and Fentanyl in Infusion Solutions. Journal of Pain and Symptom Management. 1998;16(1):52–8. 10.1016/S0885-3924(98)00024-4. 9707657

[16] Allwood MC. The stability of diamorphine alone and in combination with anti-emetics in plastic syringes. Palliative Medicine. 1991;5(4):330–3. 10.1177/026921639100500409

[17] Grassby PF, Hutchings L. Drug combinations in syringe drivers: the compatibility and stability of diamorphine with cyclizine and haloperidol. Palliative Medicine. 1997;11(3):217–24. 10.1177/026921639701100306 9205655

[18] Barcia E, Reyes R, Azuara ML, Sánchez Y, Negro S. Stability and compatibility of binary mixtures of morphine hydrochloride with hyoscine-n-butyl bromide. Supportive Care in Cancer. 2005;13(4):239–45. 10.1007/s00520-004-0719-x 15798917

[19] Barcia E, Reyes R, Luz Azuara M, Sánchez Y, Negro S. Compatibility of haloperidol and hyoscine-N-butyl bromide in mixtures for subcutaneous infusion to cancer patients in palliative care. Supportive Care in Cancer. 2003;11(2):107–13. 10.1007/s00520-002-0415-7 12560939

[20] Collins AJ, Abethell JA, Holmes SG, Bain R. Stability of diamorphine hydrochloride with haloperidol in prefilled syringes for continuous subcutaneous administration. Journal of Pharmacy and Pharmacology. 1986;38(S12):51P–P. 10.1111/j.2042-7158.1986.tb14280.x2869127

[21] Donnelly RF. Physical Compatibility and Chemical Stability of Ketamine–Morphine Mixtures in Polypropylene Syringes. The Canadian Journal of Hospital Pharmacy. 2009;62(1):28–33. 22478862 10.4212/cjhp.v62i1.114 PMC2826901

[22] Ensom MHH, Decarie D, Leung K, Montgomery C. Stability of Hydromorphone–Ketamine Solutions in Glass Bottles, Plastic Syringes, and IV Bags for Pediatric Use. The Canadian Journal of Hospital Pharmacy. 2009;62(2):112–8. 22478876 10.4212/cjhp.v62i2.439 PMC2826933

[23] Fielding H, Kyaterekera N, Skellern GG, Tettey JN, McDade JR, Msuya Z, et al The compatibility and stability of octreotide acetate in the presence of diamorphine hydrochloride in polypropylene syringes. Palliative Medicine. 2000;14(3):205–7. 10.1191/026921600666997498 . 10858828

[24] Hor MMS, Chan SY, Yow KL, Lim LY, Chan E, Ho PC. Stability of admixtures of pethidine and metoclopramide in aqueous solution, 5% dextrose and 0·9% sodium chloride. Journal of Clinical Pharmacy and Therapeutics. 1997;22(5):339–45. 10.1046/j.1365-2710.1997.00113.x 19160718

[25] Jäppinen A, Kokki H, Naaranlahti TJ, Rasi AS. Stability of buprenorphine, haloperidol and glycopyrrolate mixture in a 0.9% sodium chloride solution. Pharmacy World and Science. 1999;21(6):272–4. 10.1023/a:1008771621812 10658237

[26] Nassr S, Brunet M, Lavoie P, Brazier JL. HPLC–DAD METHOD FOR STUDYING THE STABILITY OF SOLUTIONS CONTAINING MORPHINE, DEXAMETHASONE, HALOPERIDOL, MIDAZOLAM, FAMOTIDINE, METOCLOPRAMIDE, AND DIMENHYDRINATE. Journal of Liquid Chromatography & Related Technologies. 2001;24(2):265–81. 10.1081/JLC-100001487

[27] Nassr S, Dubuc MC, Lavoie P, Brazier JL. HPLC-DAD methods for studying the stability of solutions containing hydromorphone, ketorolac, haloperidol, midazolam, famotidine, metoclopramide, dimenhydrinate, and scopolamine. Journal of Liquid Chromatography & Related Technologies. 2003;26(17):2909–29.

[28] Negro S, Rendon AL, Azuara ML, Sánchez Y, Fernández-Carballido A, Barcia E. Compatibility and Stability of Furosemide and Dexamethasone Combined in Infusion Solutions. Arzneimittelforschung. 2006;56(10):714–20. 10.1055/s-0031-1296778 17225568

[29] Negro S, Salama A, Sanchez Y, Azuara ML, Barcia E. Compatibility and stability of tramadol and dexamethasone in solution and its use in terminally ill patients. J Clin Pharm Ther. 2007;32(5):441–4. Epub 2007/09/19. 10.1111/j.1365-2710.2007.00839.x . 17875108

[30] Peterson GM, Miller KA, Galloway JG, Dunne PF. Compatibility and stability of fentanyl admixtures in polypropylene syringes. Journal of Clinical Pharmacy and Therapeutics. 1998;23(1):67–72. 10.1046/j.1365-2710.1998.00141.x 9756114

[31] Targett PL, Keefe PA, Merridew CG. Compatibility and Stability of Drug Adjuvants and Morphine Tartrate in 10 mL Polypropylene Syringes. The Australian Journal of Hospital Pharmacy. 1997;27(3):207–12. 10.1002/jppr1997273207

[32] Watson DG, Lin M, Morton A, Cable CG, McArthur DA. Compatibility and stability of dexamethasone sodium phosphate and ketamine hydrochloride subcutaneous infusions in polypropylene syringes. J Pain Symptom Manage. 2005;30(1):80–6. Epub 2005/07/27. 10.1016/j.jpainsymman.2005.01.018 . 16043011

[33] Destro M, Ottolini L, Vicentini L, Boschetti S. Physical compatibility of binary and ternary mixtures of morphine and methadone with other drugs for parenteral administration in palliative care. Support Care Cancer. 2012;20(10):2501–9. Epub 2012/01/19. 10.1007/s00520-011-1363-x . 22252547

[34] Ensom M, Decarie D, Leung K, Montgomery C. Stability of Hydromorphone–Ketamine Solutions in Glass Bottles, Plastic Syringes, and IV Bags for Pediatric Use. Can J Hosp Pharm. 2009;62(2):112–8. 22478876 10.4212/cjhp.v62i2.439 PMC2826933

[35] Fielding H, Kyaterekera N, Skellern GG, Tettey JN, McDade JR, Msuya Z, et al The compatibility and stability of octreotide acetate in the presence of diamorphine hydrochloride in polypropylene syringes. Palliat Med. 2000;14(3):205–7. Epub 2000/06/20. 10.1191/026921600666997498 . 10858828

[36] Peterson GM, Khoo BHC, Galloway JG, P JA.. Preliminary study of the stability of midazolam in polypropylene syringes. Australian Journal of Hospital Pharmacy. 1991;21:115–8.

[37] Trissel LA. Handbook on Injectable Drugs. 16 ed Bethesda, MD: American Society of Health System Pharmacists; 2010.

[38] Lach JL, Patel DM, Blaug SM. The chromatographic separation of morphine from its degradation products. Journal of the American Pharmaceutical Association. 1956;45(9):611–3. 10.1002/jps.3030450909 13357366

[39] National Institute for Health and Care Excellence (NICE). Palliative care for adults: strong opioids for pain relief (CG140)2012 11/07/2016:[9 p.]. https://www.nice.org.uk. 32208569

