# Supplementary material for: The current evidence base for the feasibility of 48-hour continuous subcutaneous infusions (CSCIs): A systematically-structured review
Source: PLoS One. 2018 Mar 14;13(3):e0194236. doi: 10.1371/journal.pone.0194236 (PMC5851608; doi:10.1371/journal.pone.0194236)
Supplement: S1 Table — Summary table or articles included in this review. (PDF) [file pone.0194236.s001.pdf]

Study, Year and Country	Study Design	Methods used to investigate stability/compatibility	Analysis	Drugs combinations investigated	Time periods at which admixtures investigated
Good et al; 2004; Australia[12]	Qualitative and Quantitative analysis of solutions stored in polypropylene syringes at room temperature (22-26ºC) and body temperature (36-39ºC)	HPLC to confirm drug concentration/degradation<br>Visual inspection for colour change/ precipitation/ evaporation<br>pH	Two-way analysis of variance with replicates	Midazolam hydrochloride and Dexamethasone sodium phosphate	Visual inspection and HPLC investigation at t=0, t=24 and t=48 hours.<br>pH tested at t=0 and t=48 hours.
Wilson et al; 1998; Australia[14]	Qualitative and Quantitative analysis of solutions stored in polypropylene syringes at 5ºC, 22ºC and 38ºC	HPLC to confirm drug concentration/degradation<br>Visual inspection for colour change/ precipitation/ evaporation<br>pH	Two-way analysis of variance with replicates	Fentanyl citrate and Midazolam hydrochloride	Visual inspection and HPLC investigation at t=0, t=24, 48, 96, and 168 hours.<br>pH tested at t=0 and t=168 hours.
Negro et al; 2006; Spain[13]	Qualitative and Quantitative analysis of solutions stored in polypropylene syringes at 25±0.5ºC	HPLC to confirm drug concentration/degradation<br>Visual inspection for colour change/ precipitation/ evaporation<br>pH	Two-way analysis of variance with replicates	Morphine hydrochloride + Haloperidol lactate + Hyoscine-*N*-Butylbromide	Visual inspection and HPLC investigation at t=0, t=5, 7, 15 days<br>pH tested at t=0 and t=15 days
Peterson et al; 1998; Australia[29]	Qualitative and Quantitative analysis of solutions stored in polypropylene syringes at 32.1±1ºC	HPLC to confirm drug concentration/degradation<br>Visual inspection for colour change/ precipitation/ evaporation<br>pH	Mean of triplicate results and standard deviations	Fentanyl citrate + Hyoscine-*N*-butylbromide + Midazolam hydrochloride<br><br>Fentanyl citrate + Metoclopramide hydrochloride + Midazolam hydrochloride	Visual inspection, pH and HPLC investigation at t=0,2,3,7,10 days
Barcia et al; 2003; Spain[18]	Qualitative and Quantitative analysis of solutions stored in polypropylene syringes at 4ºC and 25ºC	HPLC to confirm drug concentration/degradation<br>Visual inspection for colour change/ precipitation/ evaporation<br>pH	Mean of quadruplicate results and standard deviations	Hyoscine-*N*-butylbromide + Haloperidol lactate	Visual inspection, pH and HPLC investigation at t=0, t=5, 7, 15 days
Targett et al; 1997; Australia[30]	Qualitative and Quantitative analysis of solutions stored in polypropylene syringes at either 4-8ºC or 21-23 ºC	HPLC to confirm drug concentration/degradation<br>Visual inspection for colour change/ precipitation/ evaporation<br>pH	Mean of 3 syringes tested in duplicate and standard deviations	Morphine tartrate + Dexamethasone sodium phosphate + Droperidol + Hyoscine-*N*-butylbromide + Midazolam hydrochloride	Visual inspection, pH and HPLC investigation of A at t=0, 2, 5, 8, 11, 12, 14 days<br><br>Visual inspection, pH and HPLC investigation of B at t=0, 2, 5, 7, 9, 14 days
Fielding et al; 2000, UK[34]	Qualitative and Quantitative analysis of solutions stored in polypropylene syringes at 37°C	HPLC to confirm drug concentration/degradation<br>Visual inspection for colour change/ precipitation/ evaporation<br>pH	Mean of triplicate results and standard deviations	Diamorphine hydrochloride + Octreotide acetate	Visual inspection and pH at t=0 and t=48 hours<br>HPLC investigation at t=2,4,6,8,24,30, 48 hours
Negro et al; 2007, Spain[28]	Qualitative and Quantitative analysis of solutions stored in polypropylene syringes at 25°C	HPLC to confirm drug concentration/degradation<br>Visual inspection for colour change/ precipitation/ evaporation<br>pH	Mean of quintuplicate results and standard deviations	Tramadol hydrochloride and Dexamethasone sodium phosphate	Visual inspection, pH and HPLC investigation at t=1,3 and 5 days
Destro et al; 2012, Italy[32]	Qualitative and Quantitative analysis of solutions stored in polypropylene syringes at 25°C	HPLC to confirm drug concentration/degradation<br>Visual inspection for colour change/ precipitation/ evaporation<br>pH	None	Morphine hydrochloride and Ketorolac tromethamine	Visual inspection, pH and HPLC investigation at t=0 and 48 hours

Grassby et al; 1991, UK[16]	Qualitative and Quantitative analysis of solutions stored in polypropylene syringes at 22°C	HPLC to confirm drug concentration/degradation<br>Visual inspection for colour change/ precipitation/ evaporation<br>pH	Regression analysis	Diamorphine hydrochloride + Cyclizine lactate<br><br>Diamorphine hydrochloride + Haloperidol lactate<br><br>Diamorphine hydrochloride + Cyclizine lactate + Haloperidol lactate	Visual inspection, pH and HPLC investigation at t=0, 1, 2 and 7 days
Negro et al; 2006; Spain[27]	Qualitative and Quantitative analysis of solutions stored in polypropylene syringes at 4°C 25°C	HPLC to confirm drug concentration/degradation<br>Visual inspection for colour change/ precipitation/ evaporation<br>pH	Mean of quintuplicate results and standard deviations	Furosemide sodium + Dexamethasone sodium phosphate	Visual inspection, pH and HPLC investigation at t=0,5 and 15 days
Nassr et al; 2003, Canada[26]	Qualitative and Quantitative analysis of solutions stored in polypropylene syringes at 4°C and 25°C	HPLC to confirm drug concentration/degradation<br>Visual inspection for colour change/ precipitation/ evaporation	Mean of quintuplicate results	Hydromorphone hydrochloride + midazolam hydrochloride + Famotidine<br><br>Hydromorphone hydrochloride + Metoclopramide hydrochloride + Haloperidol lactate<br><br>Hydromorphone hydrochloride + Ketorolac tromethamine + Metoclopramide hydrochloride + Famotidine<br><br>Hydromorphone hydrochloride + Dimenhydrinate + Haloperidol lactate + Famotidine + Hyoscine hydrobromide	Visual inspection and HPLC investigation at t=0, 4, 8, 12, 24, 48, 72 and 96 hours
Nassr et al; 2001, Canada [25]	Qualitative and Quantitative analysis of solutions stored in polypropylene syringes at 4°C and 25°C	HPLC to confirm drug concentration/degradation<br>Visual inspection for colour change/ precipitation/ evaporation	Mean of quintuplicate results	Morphine sulphate + Dexamethasone sodium phosphate + Octreotide acetate<br><br>Morphine sulphate + Dexamethasone sodium phosphate + Haloperidol lactate<br><br>Morphine sulphate + Octreotide acetate + Haloperidol lactate + Midazolam hydrochloride + Famotidine<br><br>Morphine sulphate + Haloperidol lactate + Famotidine + Metoclopramide hydrochloride<br><br>Morphine sulphate + Octreotide acetate + Haloperidol lactate + Famotidine + Metoclopramide hydrochloride + Dimenhydrinate	Visual inspection and HPLC investigation at t=0, 4, 8, 12, 24, 48, 72 and 96 hours
Donnelly; 2009; Canada[20]	Qualitative and Quantitative analysis of solutions stored in polypropylene syringes at 5°C and 23°C	HPLC to confirm drug concentration/degradation<br>Visual inspection for colour change/ precipitation/ evaporation<br>pH	Mean of triplicate results and standard deviations	Morphine sulphate + Ketamine hydrochloride	Visual inspection, pH and HPLC investigation at t=7, 14, 28, 56 and 91 days
Ensom et al; 2009; Canada[33]	Qualitative and Quantitative analysis of solutions stored in polypropylene syringes at 25°C	HPLC to confirm drug concentration/degradation<br>Visual inspection for colour change/ precipitation/ evaporation<br>pH	Mean of triplicate or quadruplicate results and standard deviations	Hydromorphone hydrochloride + Ketamine hydrochloride	Visual inspection, pH and HPLC investigation at t=0, 1,2, 3 and 7 days

Watson et al; 2005; UK[31]	Qualitative and Quantitative analysis of solutions stored in polypropylene syringes at 4°C, 23°C and 37°C	HPLC to confirm drug concentration/degradation<br>Visual inspection for colour change/ precipitation/ evaporation<br>pH	Mean of sextuplicate results and standard deviations	Dexamethasone sodium phosphate + Ketamine Hydrochloride	Visual inspection, pH and HPLC investigation at t=0, 2, 4, 8, 24, 48, 96 and 192 hours
Hor et al; 1997; Singapore[23]	Qualitative and Quantitative analysis of solutions stored in polypropylene syringes at 32°C	HPLC to confirm drug concentration/degradation<br>Visual inspection for colour change/ precipitation/ evaporation<br>pH	Mean of duplicate results and standard deviations	Pethidine hydrochloride + Metoclopramide hydrochloride	Visual inspection, pH and HPLC investigation at t=0, 0.5, 1, 2, 4, 6, 8, 24, 32 and 48 hours
Barcia et al; 2005; Spain[17]	Qualitative and Quantitative analysis of solutions stored in polypropylene syringes at 4ºC and 25ºC	HPLC to confirm drug concentration/degradation<br>Visual inspection for colour change/ precipitation/ evaporation<br>pH	Mean of triplicate results and standard deviations	Morphine hydrochloride + Hyoscine-*N*-Butylbromide	Visual inspection, pH and HPLC investigation at t=0, t=5, 7, 15 days
Jäppinen et al; 1999; Finland[24]	Qualitative and Quantitative analysis of solutions stored in polypropylene syringes and polyvinylchloride cassettes at 4ºC, 21ºC and 36ºC	HPLC to confirm drug concentration/degradation<br>Visual inspection for colour change/ precipitation/ evaporation<br>pH<br>Membrane filtration for microbiological essay	Mean of quadruplicate results and standard deviations	Buprenorphine hydrochloride + Haloperidol lactate + Glycopyrronium bromide	Visual inspection and HPLC investigation at t=0, t=1, 2, 3, 9 16 and 30 days<br>pH tested at t=0 and t=30 days<br>Microbiological assay at t=12 hours and t=30 days
Allwood; 1991; UK[15]	Qualitative and Quantitative analysis of solutions stored in polypropylene syringes at ambient temperature 18-22ºC	HPLC to confirm drug concentration/degradation<br>Visual inspection for colour change/ precipitation/ evaporation<br>pH	Regression analysis of the mean of 2 syringes tested in triplicate	Diamorphine hydrochloride + Haloperidol lactate<br><br>Diamorphine hydrochloride + Cyclizine lactate	Periods at which inspections/ analysis performed not specified by article
Collins et al; 1990; UK[19]	Qualitative and Quantitative analysis of solutions stored in polypropylene syringes at ambient temperature (22-24°C) and under refrigeration (4-8°C)	HPLC to confirm drug concentration/degradation<br>Visual inspection for colour change/ precipitation/ evaporation	Mean of quadruplicate results and standard deviations	Diamorphine hydrochloride + Haloperidol lactate	Visual inspection and HPLC analysis performed on day 7 only.
