# Supplementary material for: The current evidence base for the feasibility of 48-hour continuous subcutaneous infusions (CSCIs): A systematically-structured review
Source: PLoS One. 2018 Mar 14;13(3):e0194236. doi: 10.1371/journal.pone.0194236 (PMC5851608; doi:10.1371/journal.pone.0194236)
Supplement: S1 File — Full text assessment form used, adapted from Hawker et al, 2002. (PDF) [file pone.0194236.s002.pdf]

Full Text Assessment Form

Author(s): ___________________________________ **Year of Publication:** ___________

Abbreviated Title: ____

____

Assessor: ____________________ **Date Assessed:** _______________

Study design:

- ☐ Quantitative
- ☐ Qualitative
- ☐ Combination

Location of study: _____________

Sample Size: _________________

Aim:

Research Question(s)/Hypothesis (If Any):

Method and Analysis:

Adapted from Hawker S, Payne S, Kerr C, Hardey M, Powell J. Appraising the evidence: reviewing disparate data systematically. Qual Health Res. 2002;12. doi: 10.1177/1049732302238251.

Results:

Drugs Involved and Concentrations:

Compatibility:

Stability (How assessed):

Conclusions, Comments and Issues Raised:

Adapted from Hawker S, Payne S, Kerr C, Hardey M, Powell J. Appraising the evidence: reviewing disparate data systematically. Qual Health Res. 2002;12. doi: 10.1177/1049732302238251.
