# Supplementary material for: The current evidence base for the feasibility of 48-hour continuous subcutaneous infusions (CSCIs): A systematically-structured review
Source: PLoS One. 2018 Mar 14;13(3):e0194236. doi: 10.1371/journal.pone.0194236 (PMC5851608; doi:10.1371/journal.pone.0194236)
Supplement: S2 File — Poster presented by author at the APM Supportive and Palliative Care Conference 2017 and referenced in this review. (PDF) [file pone.0194236.s003.pdf]

# Challenging the pressure on NHS resources: could 48-hour continuous subcutaneous infusions (CSCIs) help? A systematically -structured review of the current evidence base.

James Baker[1,2], Andrew Dickman[1,2], Stephen Mason[2], Paul Skipper[1], Jenny Schneider[3], John Ellershaw[1,2]

*[1]Royal Liverpool and Broadgreen University Hospitals NHS Trust, Liverpool, UK [2]Marie Curie Palliative Care Institute Liverpool, University of Liverpool, Liverpool, UK [3]The University of Newcastle, Newcastle, Australia*

The Marie Curie Palliative Care Institute LIVERPOOL

*Excellence in Care for the Dying*

## Background

The majority of patients express a preference to die at home, yet the most commonly recorded place of death is hospital; in 2012, 36.7% of deaths in Liverpool occurred in the person's usual place of residence. With an ageing population, NHS resources will be placed under increasing pressure to meet the needs and care preferences of chronically ill patients[1,2].

Innovative approaches to existing therapies are one way to improve care and maximise service delivery. For example, the ability to deliver prescribed medication by CSCI over 48 hours may have numerous benefits in both patient care and health service resource utilisation: current practice limits infusion time to a maximum of 24 hours due to available chemical and microbiological stability data.

## Aims

To examine and present the evidence on stability of 48 hour multiple-drug syringes/CSCIs in current clinical practice.

## Methods

Three electronic databases (CINAHL, EMBASE and MEDLINE) and grey literature were systematically searched using PRISMA Guidiance. Studies published in English reporting empirical data on the chemical or microbiological stability of continuous subcutaneous infusions or solutions stored in polypropylene syringes, were included.

**Figure 1: PRISMA flowchart**

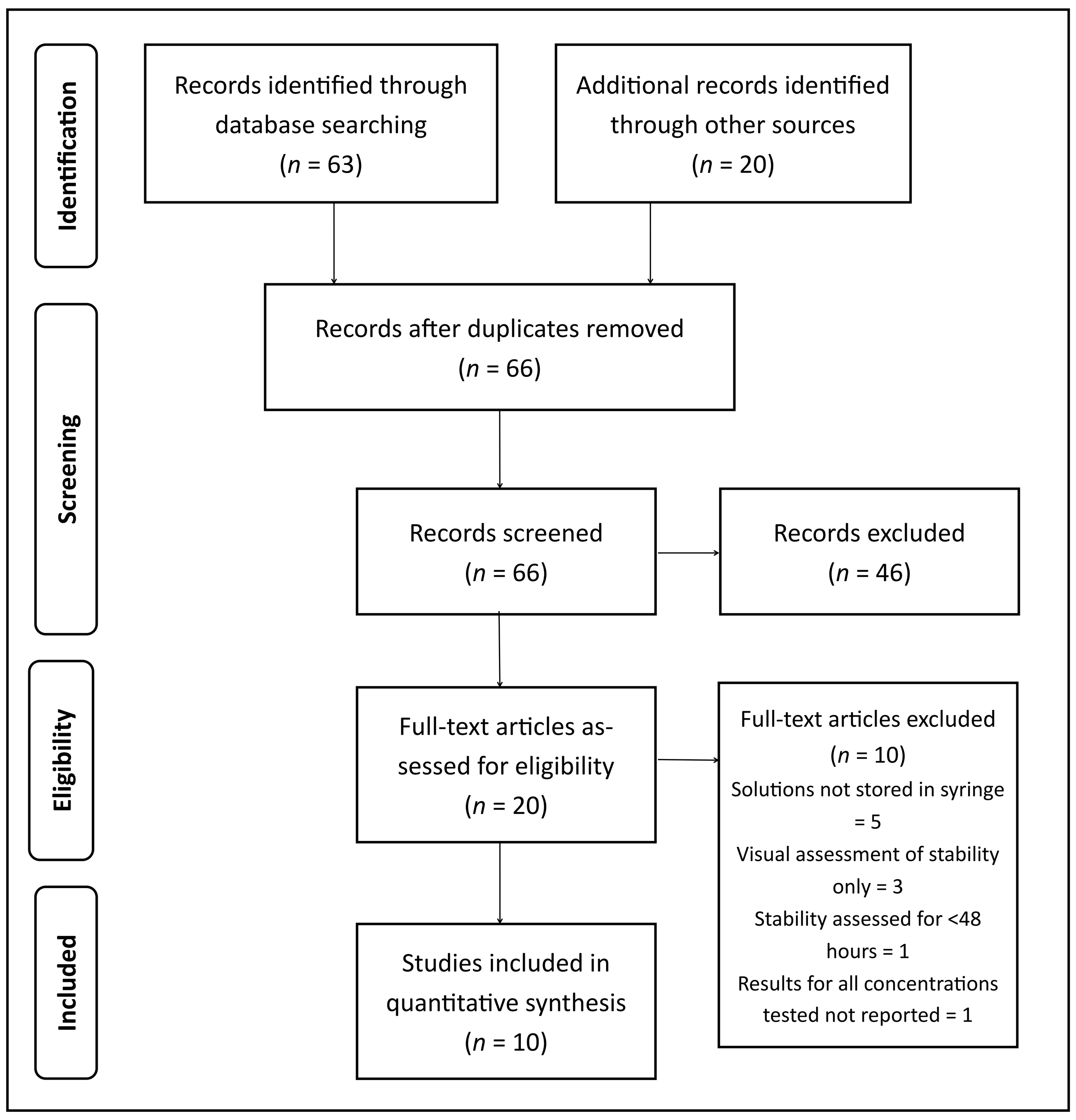


## Results

Chemical compatibility and stability of 51 different combinations of 12 drugs were reported across the ten studies included in this review (Table 1). Of the 51 combinations reported, all 51 were assessed as being chemically compatible after 48 hours at ambient temperatures (20-26°C). Nine of the thirteen drugs included are regularly utilised in the prescribing of CSCIs in the United Kingdom (midazolam, dexamethasone, hyoscine-N-butylbromide, haloperidol, fentanyl, diamorphine, cyclizine, metoclopramide and glycopyrrolate).

## Results (cont.)

Midazolam appeared to be drug at greatest risk of clinically significant chemical degradation due to its pH dependent ring structure[3]. Microbiological stability was only reported for one combination.

**Table 1: Summary of drug combinations and stability reported in reviewed articles**

| Study, Year and Country | Drug combination reported | Incompatiblity observed? |
|---|---|---|
| Good et al; 2004; Australia | Midazolam hydrochloride 2.5mg + Dexamethasone sodium phosphate 4mg | No |
| | Midazolam hydrochloride 2.5mg + Dexamethasone sodium phosphate 2mg | No |
| | Midazolam hydrochloride 5mg + Dexamethasone sodium phosphate 2mg | No |
| | Midazolam hydrochloride 7.5mg + Dexamethasone sodium phosphate 2mg | Yes @ 37°C |
| Wilson et al; 1998; Australia | Fentanyl citrate 100mcg + Midazolam hydrochloride 5mg | No |
| | Fentanyl citrate 100mcg + Midazolam hydrochloride 7.5mg | No |
| | Fentanyl citrate 300mcg + Midazolam hydrochloride 5mg | No |
| | Fentanyl citrate 300mcg + Midazolam hydrochloride 7.5mg | Yes @ 37°C |
| | Fentanyl citrate 600mcg + Midazolam hydrochloride 15mg | No |
| | Fentanyl citrate 600mcg + Midazolam hydrochloride 5mg | Yes @ 37°C |
| Negro et al; 2006; Spain | Morphine hydrochloride 100mg + Haloperidol lactate 25mg + Hyoscine-N-butylbromide 300mg | No |
| | Morphine hydrochloride 100mg + Haloperidol lactate 37.5mg + Hyoscine-N-butylbromide 300mg | No |
| | Morphine hydrochloride 100mg + Haloperidol lactate 25mg + Hyoscine-N-butylbromide 400mg | No |
| | Morphine hydrochloride 100mg + Haloperidol lactate 37.5mg + Hyoscine-N-butylbromide 400mg | No |
| | Morphine hydrochloride 300mg + Haloperidol lactate 25mg + Hyoscine-N-butylbromide 300mg | No |
| | Morphine hydrochloride 300mg + Haloperidol lactate 37.5mg + Hyoscine-N-butylbromide 300mg | No |
| | Morphine hydrochloride 300mg + Haloperidol lactate 25mg + Hyoscine-N-butylbromide 400mg | No |
| | Morphine hydrochloride 300mg + Haloperidol lactate 37.5mg + Hyoscine-N-butylbromide 400mg | No |
| | Morphine hydrochloride 600mg + Haloperidol lactate 25mg + Hyoscine-N-butylbromide 300mg | No |
| | Morphine hydrochloride 600mg + Haloperidol lactate 37.5mg + Hyoscine-N-butylbromide 300mg | No |
| | Morphine hydrochloride 600mg + Haloperidol lactate 25mg + Hyoscine-N-butylbromide 400mg | No |
| | Morphine hydrochloride 600mg + Haloperidol lactate 37.5mg + Hyoscine-N-butylbromide 400mg | No |
| Peterson et al; 1998; Australia | Fentanyl citrate 1000mcg + Hyoscine-N-butylbromide 30mg + Midazolam hydrochloride 15mg | No |
| | Fentanyl citrate 1000mcg + Metoclopramide hydrochloride 20mg + Midazolam hydrochloride 15mg | No |
| Barcia et al; 2003; Spain | Haloperidol lactate 18.75mg + Hyoscine-N-butylbromide 150mg | No |
| | Haloperidol lactate 18.75mg + Hyoscine-N-butylbromide 300mg | No |
| | Haloperidol lactate 18.75mg + Hyoscine-N-butylbromide 600mg | No |
| | Haloperidol lactate 37.5mg + Hyoscine-N-butylbromide 150mg | No |
| | Haloperidol lactate 37.5mg + Hyoscine-N-butylbromide 300mg | No |
| | Haloperidol lactate 37.5mg + Hyoscine-N-butylbromide 600mg | No |
| | Haloperidol lactate 75mg + Hyoscine-N-butylbromide 150mg | Yes, @ 4°C and 25°C |
| | Haloperidol lactate 75mg + Hyoscine-N-butylbromide 300mg | Yes, @ 4°C and 25°C |
| | Haloperidol lactate 75mg + Hyoscine-N-butylbromide 600mg | Yes, @ 4°C and 25°C |
| Barcia et al; 2005; Spain | Morphine hydrochloride 100mg + Hyoscine-N-butylbromide 200mg | No |
| | Morphine hydrochloride 100mg + Hyoscine-N-butylbromide 300mg | No |
| | Morphine hydrochloride 100mg + Hyoscine-N-butylbromide 400mg | No |
| | Morphine hydrochloride 300mg + Hyoscine-N-butylbromide 200mg | No |
| | Morphine hydrochloride 300mg + Hyoscine-N-butylbromide 300mg | No |
| | Morphine hydrochloride 300mg + Hyoscine-N-butylbromide 400mg | No |
| | Morphine hydrochloride 600mg + Hyoscine-N-butylbromide 200mg | No |
| | Morphine hydrochloride 600mg + Hyoscine-N-butylbromide 300mg | No |
| | Morphine hydrochloride 600mg + Hyoscine-N-butylbromide 400mg | No |
| Jäppinen et al; 1999; Finland | Buprenorphine hydrochloride 4mg + Haloperidol lactate 5mg + Glycopyrronium bromide 1.2mg | No |
| Targett et al; 1997; Australia | Morphine tartrate 400mg + Dexamethasone sodium phosphate 8mg + Droperidol 2mg + Hyoscine-N-butylbromide 20mg + Midazolam hydrochloride 8mg | Yes @ 22°C |
| | Morphine tartrate 40mg + Dexamethasone sodium phosphate 8mg + Droperidol 2mg + Hyoscine-N-butylbromide 20mg + Midazolam hydrochloride 5mg | No |
| Allwood; 1991; UK | Diamorphine hydrochloride 20mg + Haloperidol lactate 7.5mg | No |
| | Diamorphine hydrochloride 200mg + Haloperidol lactate 7.5mg | No |
| | Diamorphine hydrochloride 20mg + Cyclizine lactate 67mg | No |
| | Diamorphine hydrochloride 200mg + Cyclizine lactate 67mg | No |
| Collins et al; 1990; UK | Diamorphine hydrochloride 50mg + Haloperidol lactate 2.5mg | No |
| | Diamorphine hydrochloride 100mg + Haloperidol lactate 2.5mg | No |

## Conclusion

There is currently limited evidence for the physical, chemical and microbiological stability of solutions for continuous subcutaneous infusion over a period of 48 hours. More stability data is required before the use of 48-hour CSCIs can be evaluated for use within clinical practice. The range of temperatures at which stability is tested highlights the need for consensus on how stability/compatibility should be structured.

## Acknowledgements

This project was funded through a grant from NHS Liverpool CCG.

## References

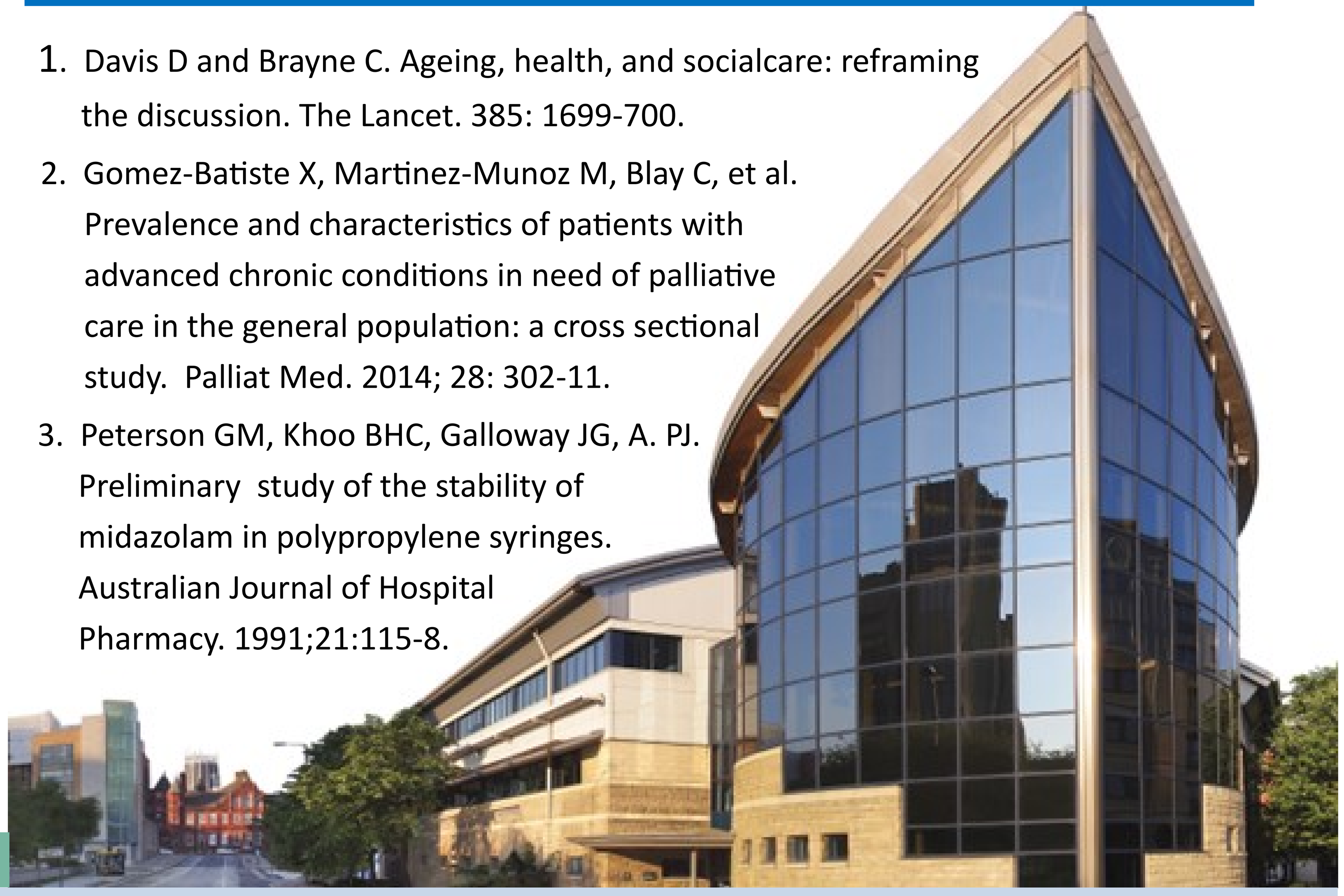

1. Davis D and Brayne C. Ageing, health, and socialcare: reframing the discussion. The Lancet. 385: 1699-700.
2. Gomez-Batiste X, Martinez-Munoz M, Blay C, et al. Prevalence and characteristics of patients with advanced chronic conditions in need of palliative care in the general population: a cross sectional study. Palliat Med. 2014; 28: 302-11.
3. Peterson GM, Khoo BHC, Galloway JG, A. PJ. Preliminary study of the stability of midazolam in polypropylene syringes. Australian Journal of Hospital Pharmacy. 1991;21:115-8.

The Royal Liverpool and Broadgreen University Hospitals NHS Trust | Marie Curie | University of Liverpool

www.mcpcil.org.uk
